# When Histiocytosis Masquerades as Mononucleosis: A Case Report

**DOI:** 10.7759/cureus.70156

**Published:** 2024-09-25

**Authors:** Mamuka Khundadze, Lali Khurtsia, Natali Shulaia, George Kandelaki

**Affiliations:** 1 Medicine, David Tvildiani Medical University, Tbilisi, GEO; 2 Infectious Diseases, Vakhtang Bochorishvili Clinic, Tbilisi, GEO; 3 Radiology, American Hospital Tbilisi, Tbilisi, GEO; 4 Infectious Diseases, New Hospitals, Tbilisi, GEO

**Keywords:** fever of unknown origin, infectious mononucleosis-like syndrome, langerhans cell histiocytosis(lch), lytic bone lesion, multiple lytic bone lesions, severe anemia

## Abstract

Langerhans cell histiocytosis (LCH) is a rare disorder predominantly affecting children and is characterized by a wide range of clinical presentations, which can make early identification of the disease difficult and result in the delay of appropriate treatment. The challenge is further compounded by the fact that diagnostic confirmation typically requires a biopsy of the bone or skin lesion, as well as immunohistochemical identification of molecular markers, which may not be readily available in all settings. This case report describes a two-year-old female who was initially misdiagnosed with infectious mononucleosis due to her non-specific presentation, highlighting the diagnostic challenges of LCH, particularly in resource-limited settings. The case highlights the importance of increasing clinical awareness of LCH and including this condition in differential diagnoses to ensure timely and appropriate management.

## Introduction

Langerhans cell histiocytosis (LCH) is a rare, neoplastic disorder that is characterized by tissue infiltration primarily by histiocytes [1]. It most frequently occurs in children aged one to three years, though it has been diagnosed across all age groups [2,3]. In a slight majority of cases, LCH affects a single site, while the remaining instances are multisystem diseases [3]. Patients with systemic disease have poorer prognosis, especially if they are less than two years old [4].

Bones are one of the most commonly involved sites in LCH, and they typically present as bone pain with lytic lesions visualized on imaging, especially in the skull and the femur. Skin is also frequently affected, which can manifest with a wide range of rashes-from maculopapular to eczematous. Beyond bone and skin, histiocytes may invade almost any part of the body, including bone marrow, spleen, and lymph nodes [3,5,6].

Bone marrow involvement causing anemia and thrombocytopenia, irrespective of leukopenia, is an especially poor prognostic factor and might significantly lower five-year survival [7]. Furthermore, conventional cytology of the bone marrow may frequently lead to overlooking the diagnosis of LCH unless immunohistochemistry targeting specific markers is employed, which presents an additional challenge for physicians [8].

Generally, diagnostic confirmation requires bone or skin lesion biopsy with histiocyte morphology being corroborated by immunohistochemical staining for markers such as CD1a, S100, and others [8,9].

The treatment of LCH varies based on the location and extent of the disease and may involve observation, surgery, radiation therapy, or the use of chemotherapeutic and immunosuppressive medications [10].

Langerhans cell histiocytosis can present in various ways, lacks pathognomonic signs or symptoms, and often initially manifests as a nonspecific illness, making it easy to overlook and delay diagnosis and treatment. This is particularly relevant for patients with a poor prognosis due to multisite involvement [11]. To illustrate these challenges, we present the case of a two-year-old female who was initially diagnosed with infectious mononucleosis due to nonspecific symptoms. However, as her condition deteriorated over weeks, the correct diagnosis of LCH was eventually made.

## Case presentation

A two-year-old female, fully vaccinated and with no significant past medical history, was admitted to our hospital in Tbilisi, Georgia, with a history of persistent fever and severe anemia lasting over a month.

Her complaints began approximately two months prior, with subfebrile temperatures averaging 37.5°C (99.5°F) that persisted for several days. After a week, the parents decided to consult a pediatrician, who performed a basic diagnostic workup, which revealed decreased hemoglobin (Hgb), mild leukocytosis, low iron levels, and elevated ferritin. The patient was prescribed iron supplementation and azithromycin and sent home with a preliminary diagnosis of bacterial upper respiratory infection.

Despite treatment with antibiotics, the fever persisted for days. Further testing revealed an elevated C-reactive protein (CRP) level, decreased Hgb, and normal liver function tests (LFTs). In addition, physical examination revealed the presence of splenomegaly, mild cervical lymphadenopathy, and subtle erythematous papular rash, mostly on the patient’s calves. At this point, she was referred to an infectious diseases' specialist who, based on clinical features, diagnosed the patient with infectious mononucleosis, discontinued azithromycin, and prescribed supportive treatment. After a few days, the rash spontaneously resolved, and the temperature returned to normal.

Less than a week following the initial improvement, the patient’s fever recurred and peaked at 39°C (102.2°F). This time, the patient was admitted to a hospital, where a diagnostic work-up revealed a further decrease in Hgb levels and red blood cell (RBC) count. She underwent bone marrow aspiration and biopsy, and after drawing blood cultures, she was started on intravenous ceftriaxone twice daily. She also received a packed red blood cell (pRBC) transfusion. Both bone marrow biopsy and blood cultures later came back negative. Due to her persistently elevated temperature, the decision was made to initiate treatment with prednisolone 10 mg twice daily. Two days after taking the steroids, the patient’s condition began to improve, pharmacotherapy was discontinued, and the patient was discharged. 

Again, approximately a week later, the patient developed high temperatures. Her pediatrician started her on 5 mg prednisolone and a cephalosporin (although the parents cannot recall the specific drug name). Despite pharmacotherapy, the patient’s condition continued to deteriorate, and due to this reason, she was now admitted to our hospital. 

At the time of admission, the patient’s temperature was 38.5°C (101.3°F), heart rate was 178 bpm, respiratory rate was 36 breaths/min, and SpO_2_ was 99% on room air. The patient was hemodynamically stable but showed significant pallor and weakness. Extremities were cool to the touch, and capillary refill time was normal. The abdomen was soft to palpation but swollen, with the liver palpable at the level of the costal arch and with evident splenomegaly. There was no rash present on the body.

Laboratory studies (Table 1) revealed normal LFTs, elevated inflammatory markers, thrombocytopenia, and severe anemia with a hemoglobin level of 4.8 g/dL, for which the patient received a pRBC transfusion. Epstein-Barr virus (EBV) IgG was positive, and EBV IgM, heterophile antibody test, and Epstein-Barr virus nuclear antigen antibody all came back negative.

**Table 1:** Relevant laboratory findings on the day of admission at our hospital and on day two. RBC: red blood cells, Hgb: hemoglobin, Hct: hematocrit: MCV: mean corpuscular volume, MCH: mean corpuscular hemoglobin, MCHC: mean corpuscular hemoglobin concentration, RDW: red cell distribution width, PLT: platelets, WBC: white blood cells, ESR: erythrocyte sedimentation rate, CRP: C-reactive protein, LDH: lactate dehydrogenase.

Parameter	Day of admission	Second day	Reference range
RBC	2.14	3.13	3.9-5.3 million cells/µL
Hgb	4.8	6.8	10.5-13.5 g/dL
Hct	14.8	21.3	33%-40%
MCV	69.2	68.1	70-86 fL
MCH	22.4	21.7	23-31 pg/cell
MCHC	32.4	31.9	32-36 g/dL
RDW	24.3	20.8	12%-14%
PLT	109,000	85,000	150,000-450,000 cells/µL
WBC	13,930	10,440	5,000-15,500 cells/µL
Neutrophils	62	62.4	20%-40%
Lymphocytes	29.3	27.2	50%-70%
Monocytes	7.5	7.7	2%-8%
Eosinophils	0.4	2.2	1%-4%
Basophils	0.3	0.5	0.5%-1%
Reticulocytes	N/A	1.5	0.8%-1.5%
ESR	N/A	73	10-20 mm/h
CRP	126.65	N/A	<10 mg/L
Ferritin	387.40	N/A	7-140 ng/m
Procalcitonin	1.70	N/A	<0.5 ng/mL
LDH	N/A	758	150-380 U/L

The abdominal ultrasound measured the spleen at 140 mm in length (normal range for 1 to 2.5-year-olds is less than 104 mm), confirming splenomegaly. Based on the patient's history, clinical presentation, and laboratory results, a blood sample was drawn for additional testing, and antibiotic therapy with cefepime was initiated.

On the second day of admission, the patient continued to experience hectic fever, with temperatures reaching 40.1°C (104.18°F) and periodic profuse sweating, particularly at night. Single pale petechiae were noted in the neck and groin regions. Meningeal signs were absent. The abdomen was distended and swollen, with pronounced splenomegaly. Complete blood count (CBC) showed an Hgb level of 6.8 g/dL, decreased hematocrit (21.3%), and worsening thrombocytopenia (Table 1). Due to her clinical condition and elevated inflammatory markers, vancomycin was added to the ongoing antibiotic therapy.

On the same day, rheumatology and infectious diseases were consulted, and it was decided to initiate pulse therapy with IV methylprednisolone at 30 mg/kg alongside the ongoing antibiotic treatment. Further management would be determined based on the test results.

**Figure 1:**
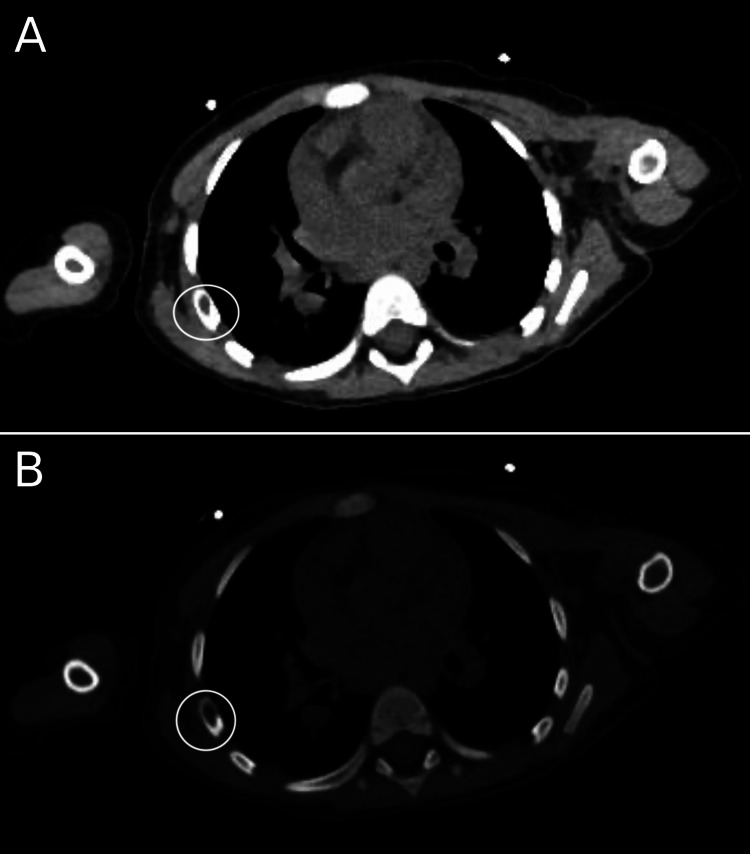
CT scan showing a lytic lesion in the right fifth rib.

**Figure 2:**
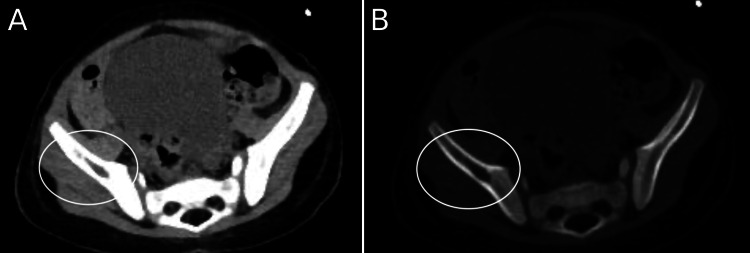
CT scan showing 1 cm lytic lesion in the right iliac wing.

**Figure 3:**
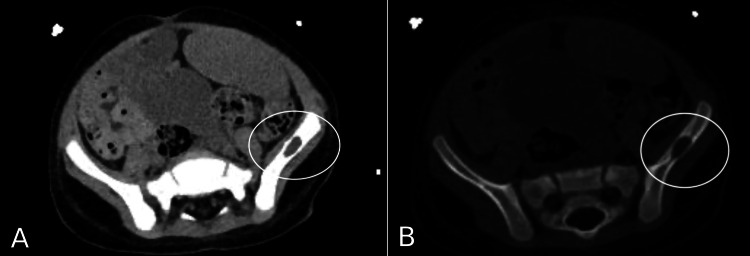
CT scan showing 1.5 cm lytic lesion in the left iliac wing.

CT scan of the thorax and abdomen done on the second day of admission which, alongside splenomegaly (longitudinal size 17.5 cm) and mild abdominopelvic lymphadenopathy, revealed the presence of lytic bone lesions in the right fifth rib (Figure 1), right iliac bone (Figure 2), and left iliac wing (Figure 3).

**Figure 4:**
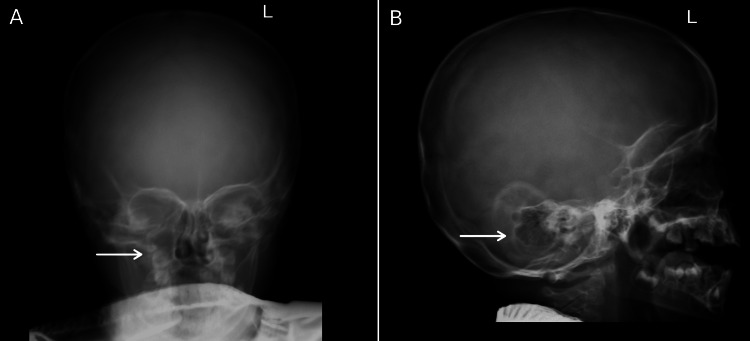
X-rays of the skull showing multiple oval-shaped destructive foci in the bones with unclear contours.

In addition, a head X-ray done on the same day revealed the presence of multiple oval-shaped destructive foci with unclear contours in the skull bones (Figure 4).

Antinuclear antibody (ANA), anti-ds-DNA, and rheumatoid factor (RF) all came back negative. Infectious or rheumatologic conditions were deemed unlikely, and hematology-oncology was consulted. Due to the presence of anemia, thrombocytopenia, exanthematous rash, splenomegaly, lymphadenopathy, and lytic bone lesions, a preliminary diagnosis of histiocytosis was made. The patient was referred to a more specialized facility for diagnostic confirmation and appropriate management.

## Discussion

Langerhans cell histiocytosis (LCH) is a rare disorder predominantly affecting children, and its diverse clinical manifestations often overlap with those of other disorders, posing significant diagnostic challenges. This case highlights the difficulties associated with diagnosing LCH, particularly within the pediatric population. 

Our patient initially presented with fever, lymphadenopathy, and splenomegaly, which led to a misdiagnosis of infectious mononucleosis accompanied by anemia attributed to iron deficiency. Such misdiagnoses are common in LCH due to the absence of pathognomonic signs and the broad differential diagnosis that includes more prevalent infections and hematologic disorders [11].

The use of antibiotics, classically ampicillin or amoxicillin, in patients with infectious mononucleosis is known to occasionally result in drug eruptions. Although not common, azithromycin can also cause a rash through mechanisms that remain unclear, and the presence of such a finding did not necessarily rule out the diagnosis of mononucleosis [12].

Later on, the decision to perform a bone marrow aspiration and biopsy was driven by the patient's persistent fever, splenomegaly and worsening anemia, raising concerns about potential hematologic malignancies or bone marrow involvement by an infiltrative process like LCH. However, unless immunohistochemistry is used to identify molecular markers indicative of histiocytosis (which would be the case if LCH specifically was also being considered), it can often yield negative results, as was observed in this case, further complicating diagnosis [8, 9].

Finally, the detection of bone lesions on CT and X-ray (Figures 1-4), and the eventual diagnosis of LCH through these radiologic studies underscores the critical role of early imaging in such complex cases. While lytic bone lesions, for example, may not be pathognomonic, their discovery turned out to be invaluable in the context of the overall clinical picture for narrowing down potential diagnoses.

## Conclusions

The case emphasizes the importance of considering LCH in the differential diagnosis when patients with nonspecific symptoms fail to sustain improvement on initial treatments and also illustrates the limitations of conventional diagnostic approaches, such as the reliance on clinical features and basic laboratory tests, which may not be sufficient to identify LCH early in its course. Imaging techniques, including CT scans, were crucial in detecting the characteristic lytic bone lesions that ultimately lead to the correct diagnosis. Timely diagnosis is especially critical for patients who have poor prognostic factors, such as multisystem disease, splenic involvement, and hematologic abnormalities, which was the case with our two-year-old patient. This emphasizes the need for a high index of suspicion and the use of appropriate diagnostic modalities in similar clinical scenarios.
